# Insights into Animal and Plant Lectins with Antimicrobial Activities

**DOI:** 10.3390/molecules20010519

**Published:** 2015-01-05

**Authors:** Renata de Oliveira Dias, Leandro dos Santos Machado, Ludovico Migliolo, Octavio Luiz Franco

**Affiliations:** 1 SInova, Programa de Pós Graduação em Biotecnologia, Universidade Católica Dom Bosco, 79117-900 Campo Grande, MS, Brazil; E-Mails: diasrdo@gmail.com (R.O.D.); leomachadovet@gmail.com (L.S.M.); ludovico.migliolo@gmail.com (L.M.); 2 Centro de Análises Proteômicas e Bioquímicas, Pós-Graduação em Ciências Genômicas e Biotecnologia, Universidade Católica de Brasília, 70790-100 Brasília, DF, Brazil

**Keywords:** lectin, carbohydrate, antimicrobial, animal defense, plant defense

## Abstract

Lectins are multivalent proteins with the ability to recognize and bind diverse carbohydrate structures. The glyco -binding and diverse molecular structures observed in these protein classes make them a large and heterogeneous group with a wide range of biological activities in microorganisms, animals and plants. Lectins from plants and animals are commonly used in direct defense against pathogens and in immune regulation. This review focuses on sources of animal and plant lectins, describing their functional classification and tridimensional structures, relating these properties with biotechnological purposes, including antimicrobial activities. In summary, this work focuses on structural-functional elucidation of diverse lectin groups, shedding some light on host-pathogen interactions; it also examines their emergence as biotechnological tools through gene manipulation and development of new drugs.

## 1. Introduction

Lectins are a complex group of proteins and/or glycoproteins of non-immune origin, possessing at least one non-catalytic domain which binds reversibly and specifically to monosaccharides, oligosaccharides and glycoconjugates. These proteins have also been named agglutinins or hemagglutinins and are found as monomers, homo- and heterodimers, as well as homo- and heterotetramer molecules, and they are widely distributed in Nature. Lectins are ubiquitous proteins and have been isolated from viruses, fungi, bacteria, invertebrates, unicellular organisms, animals and plants [1,2].

The lectin binding sites on the carbohydrate, called the carbohydrate recognition domain (CRD), promote specific recognition in accordance with the key-lock model [3]. Besides the CRD, which is highly conserved in each type of lectin [4], specificity occurs throughout a series of weak chemical interactions [5,6,7]. The diversity of biological activities conferred by lectin*-*carbohydrate binding, as well as the molecular structure and specificity of lectins, means that these proteins form a large and heterogeneous group [1,8,9]. Lectins are considered as proteins with an estimated size from 60 to 400 kDa [10]. However, some antimicrobial peptides present lectin-like activity, *i.e.*, ability to recognize and bind carbohydrate-containing surface molecules [11,12] and sometimes are also considered as lectins [13].

Although plant lectins have been widely explored, the presence of lectins in animal sources has also been observed [14]. The roles of lectins include endocytosis and intracellular transport of vector glycoprotein mechanisms [15,16], induction of apoptosis in tumoral cells [17,18,19], blocking of HIV infection [20]; regulation of bacterial cell adhesion and migration [21] and control of protein levels in the blood [22]. Lectins are also known to play important roles in the immune system by recognizing carbohydrates that are found exclusively in pathogens, or that are inaccessible in host cells. The high diversity in the functions of lectins opens new prospects for their biotechnological use in multiple fields, including human and animal health as well as agribusiness. This work focuses on a review of plant and animal lectin distribution, function and structural characterization, highlighting their functional role in host defense and their further application in the development of biotechnological tools.

## 2. Plant Lectins

Lectins have been found in many plant groups, including mono- and dicotyledons, but most frequently they have been detected in Leguminoseae and Euphorbiaceae. Lectins are distributed in various tissues such as bark, bulb, fruit, latex, leaf, nodule, whole plant, phloem sap, rhizome, root, seed, stem, tissue culture, tuber, flowers and ovaries, and they have different cellular localizations and molecular properties [1,2]. Many plants contain lectins, including different food crops such as wheat, rice, potato, tomato, soybean and bean [2].

Plant lectins play an important role in defense mechanisms against the attack of microorganisms (Table 1) [1,23]. These lectins have been demonstrated to inhibit the growth of several phytopathogenic and non-pathogenic fungi [24,25,26,27,28,29,30,31]. The targets of some plant lectins are fungi that present chitin in their cell walls, resulting in inhibitory action on the growth and development of these microrganisms [1,25,27]. Plant lectins are also involved in defense against oomycetes. The role of these lectins in *Phytophora infestans* resistance is correlated with the presence of these proteins in a complex that recognizes the INF_1_ elicitor of *Phytophthora infestans*, transduces the hypersensitive response [32] and also strengthens the cell wall and plasma membrane (CW-PM) adhesion after infestation [33]. In fungal infections, different lectins are involved in *Arabidopsis thaliana* resistance [34,35,36,37]. The role of lectins in the antibacterial defense of *A. thaliana* is correlated with bacteria-mediated pattern-triggered immunity (PTI) [36] and with stomatal innate immunity, acting in the bacteria-mediated stomatal closure [37]. In the virus response, different plant lectins have been associated with the inhibition of the systemic spread of the tobacco etch virus [38] and with resistance in the primary and latter stages of *potexvirus* infection [39].

**Table 1:** Plant lectins showing antimicrobial activities.

Family	Species	Defense against	Reference
Lectin Receptor Kinases (LecRK)	*Arabidopsis thaliana*	**Fungi**: *Botrytis cinerea*, *Erysiphe cichoracearum*, *Erysiphe orontii*, *Blumeria graminis*, *Gigaspora rosea*; **Oomycete**: *Phytophthora infestans*; **Virus**: Turnip mosaic and Cabbage leaf curl; **Bacteria**: *Pseudomonas syringae*	[36,37,40,41]
	*Nicotiana benthamiana*	**Oomycete**: *P. infestans*	[32]
	*Oryza sativa*	**Fungi**: *Magnaporthe grisea*	[34]
Amaranthins	*Amaranthus viridis*	**Fungi**: *B. cincerea*, *Fusarium oxysporum*	[42]
Calreticulin/calnexin	*N. benthamiana*	**Oomycete**: *P. infestans*	[43]
	*A. thaliana*	**Bacteria**: *P. syringae*	[44]
EUL-related lectins	*O. sativa*	**Bacteria**: *Xanthomonasoryzae* pv. *oryzae*; **Fungi**: *Magnaporthe oryzae*	[45]
Jacalin-related lectins (JRLs)	*Triticum aestivum*	**Fungi**: *Fusarium graminearum*, *B. graminis* f. sp. Tritici, *B. cinerea*; ***Oomycete**: Phytophthora parasitica var nicotianae*; ***Bacteria***: *P. syringe pv tabaci*; ***Virus***: *tobacco mosaic virus (TMV)*	[46,47,48,49]
	*A. thaliana*	**Fungi**: *F. graminearum*, *B. graminis*f. sp. Tritici, *B.cinerea* **Virus**: tobacco etch virus; Plantago asiatica mosaic virus (PlAMV)	[38,39,46,50,51,52]
	*O. sativa*	**Fungi**: *M.grisea*	[52]
Nictaba-related	*A.thaliana*	**Fungi**:* A. flavus*, *Fusarium moniliforme*, *Fusarium solani*, *Rhizoctonia solani* and *Trichoderma harzianum* **Virus**: Cucurbit aphid-borne yellows virus	[50] [53]
Ricin-B	Transgenic *N. benthamiana*	**Virus**: tobacco etch virus	[54,55]

The most accepted classification of the plant lectins was the one presented by Van Damme* et al.* [2,56]. These authors divided the plant lectins into seven structurally and evolutionarily related protein families and, more recently, they included new proteins and redistributed the lectins in 12 families with different carbohydrate binding domains [56]. Recently, Lannoo and Van Damme [57] detailed seven of the best known lectin groups, dividing them into membrane-bound and soluble proteins with a lectin domain. Membrane-bound lectins include lectin receptor kinases (LecRK), and soluble proteins include amaranthins, calreticulin/calnexin, EUL-related lectins, jacalin-related lectins (JRLs), nictaba-related lectins and ricin-B lectins [57]. All of these classes were reported to be involved in antimicrobial defense (Table 1).

Lectin receptor kinases (LecRKs) are composed of two domains separated by a transmembrane region: an N-terminal extracellular lectin domain and a C-terminal cytosolic kinase domain. LecRK proteins are typically classified into three types: G-, C- and L-type [58,59], based on their lectin domain composition in the N-terminal region (*i.e*., similar to *Galanthus nivalis* agglutinin—GNA, calcium-dependent and legume-like lectins, respectively). Seventy-five LecRK genes (32 G-, 42 L- and 1 C-type) were identified in the *Arabidopsis thaliana* genome, while 173 genes (72 L-, 100 G- and 1 C-type) were identified in the rice genome [59]. Several LecRK genes were observed responding to abiotic and biotic stimuli in *Arabidopsis thaliana* [37,40,41]. The L-type LecRKs are involved in resistance to bacteria [37], oomycetes [32,33] and fungi [35].

The amaranthin lectin group encompasses the original protein found in the seeds of *Amaranthus caudatus* [60] and similar proteins from other plant species. The specificity of amaranthin for the carcinoma-associated T- and cryptic T-antigens (Thomsen-Friedenreich antigen) has made these proteins a specific carcinoma marker [61,62]. In plant defense, amaranthin showed clear effects on aphids, in which it induced significant mortality [63] and reduced the population growth when present in transgenic cotton [64] and potato [65]. Besides the well-known antiproliferation role, the lectin from *Amaranthus viridis* Linn seeds also presents antifungal activity, inhibiting the growth of the phytopathogenic fungi *Botrytis cincerea* and *Fusarium oxysporum* [42].

Moreover, there are lectins located in sub-celullar organelles. For example, the endoplasmic reticulum (ER) presents two homologue lectins with a chaperone role: the membrane-bound calnexin (CNX) and the soluble calreticulin (CRT) protein [66]. They act together with other proteins promoting folding and quality control of glycoproteins in the ER [66]. Moreover, CRT is also involved in Ca^2+^ homeostasis in plant cells [67], abiotic stress response [68,69], plant immunity [44] and resistance to nematodes [70] and bacteria [44].

The *Euonymus* lectin-like (EUL) domain is widespread in plants, indicating the universal role of this lectin group in these organisms [71,72]. However, despite their wide distribution inside plants, these proteins were only recently described as a particular lectin group [73]. This EUL domain was shown to represent a conserved structural unit of a novel family of putative carbohydrate-binding proteins [71]. Although its agglutinative function was known [74,75,76], this lectin could not be classified into any of the known lectin families due to a lack of sequence information. These proteins are located in the nucleocytoplasmic compartment of the plant cells and present different specificities for diverse carbohydrate structures, depending on the protein and species studied [45,73,77]. Moreover, these proteins can be composed of a single domain (type S) or two tandem domains (type D) [45]. Rice EUL-related lectin genes were observed as up-regulated after abiotic stress (ABA and NaCL treatment) and down-regulated in fungus (*Magnaporthe oryzae*) infection [45].

Additionally, jacalin-related lectins (JRLs) are a group of plant lectins with one or more domain that is homologous to the jacalin protein. There are two subfamilies of JRLs: galactose-binding and mannose-binding jacalins [56]. Galactose-binding jacalins reside in the vacuolar and mannose-binding in the nucleocytoplasmic compartment of the plant cell [78]. Galactose-specific JRLs have been described in the family Moraceae, whereas the mannose-specific JRLs are common in Viridiplantae [56]. The jacalin-related lectin genes have been shown to be associated with disease resistance, abiotic stress signaling, wounding, insect damage or multiple stresses [79].

Similarly to JRLS, nictaba plays a role in certain regulatory and cell signaling pathways in plants [80,81]. Nictaba is produced in the tobacco plant after exposure to the plant hormone jasmonic acid methyl ester [80] as well as after insect herbivory [82]. Jasmonates are important signaling molecules for plant responses to abiotic and biotic stresses, as well as in plant development. They regulate induced defense mechanisms in plants after insect attack and wound response in general [83]. The accumulation of nictaba is not only confined to the leaf subjected to jasmonate treatment or insect herbivory, but can also be observed in other leaves of the tobacco plant, indicating a systemic response [84].

Another lectin involved in plant defense signaling is ricin. Ricin is included in the lectin family ricin-B, which has biological activity and toxicity in plants. Ricin-B related proteins accumulate in the plant vacuole or are secreted to the extracellular space [56]. This lectin can play a role in plant defense against pathogens [55,85] and insects [86,87]. Although there are studies describing the antiviral activity of this lectin [54,55,85], its function has not yet been elucidated.

## 3. Animal Lectins

Animal lectins present an important role in host defense against microorganisms (Table 2). Their role in fungal defense is correlated with the control of host responses [88]. In antibacterial defense, lectins are correlated with pore-forming activity, which causes bacterial membrane permeabilization [89]. Some lectins present specificity for Gram-positive and not for Gram-negative bacteria, due to the inhibition of pore activity by lipopolysaccharides [89]. Moreover, lectins present a significant role in the host’s defense against pathogens, by activating antibacterial autophagy [90] and vacuole lysis [91]. In viral infections, lectins are involved in binding and inhibiting pervasion and replication [50,92,93], and detect the pathogen-associated molecular patterns (PAMPs) of the viruses [94]. In contrast, some viruses can proliferate due to the presence of lectins [95].

**Table 2:** Animal lectins showing antimicrobial activities.

Family	Species	Pathogen	Reference
Calnexin/Calreticulin	*Marsupenaeus japonicus*	**Bacteria**: *Vibrio anguillarum*	[96]
	*Branchiostoma japonicum*	**Bacteria**: *Escherichia coli*; *Staphylococcus aureus*	[97]
L-type	*Ictalurus punctatus*	**Bacteria**: *Edwardsiella ictaluri*	[98]
	*Marsupenaeus japonicus*	**Bacteria**: *Vibrio anguillarum*	[92]
	*Eriocheir sinensis*	**Bacteria**: *Staphylococcus aureus*; *Vibrio parahaemolyticus*; *Aeromonas hydrophila*	[99]
C-type	*Homo sapiens*	**Bacteria**: *Listeria monocytogenes*	[89]
Galectin	*Homo sapiens*	**Bacteria**: *Neisseria gonorrhoeae*;	[100]
	*Mus musculus*	**Fungi**: *Candida albicans*	[88,101]

The first attempt to divide the animal lectins into functional categories placed them in two main groups: the C-type (Ca^2+^-dependent) and S-type lectins (thiol-dependent) [102]. However, with the increase in the number of described sequences, this classification changed and included several new groups [14]. Animal lectins present at least 25 different types of fold, including C-type, I-type (Ig fold), P-type, β-sandwich (jelly-roll, present in gallectins), calnexin/calreticulin, ERGIC-53 (endoplasmatic reticulum-Golgi intermediate compartment-53—L-type), G-domains of the LNS family (laminin, agrin), β-trefoil, cysteine-rich domain of C-type macrophage mannose receptor, fibrinogen-like domain (ficolins), intelectins and tachylectin-5 (reviewed in [103]). Some of these lectin groups have been proposed as being involved in antimicrobial resistence (Table 2). However, until now, some animal lectins have not presented the antimicrobial response described, but are recognized as involved in other relevant activities, such as antitumoral factors [17].

Like their plant homologues (described in Section 2), the animal-source proteins calreticulin (Crt) and calnexin (Cnx) are homologous lectins that interact with synthesized glycoproteins in the endoplasmic reticulum (ER) and serve as molecular chaperones during folding and quality control [104]. In these organisms, Cnx and Crt also have important functions in phagocytosis [105] and apoptotic processes induced by ER stresses [106], as well as being related to viral infection [107,108,109].

In relation to L-type lectins (LTLs), these lectin domains have been found in leguminous plant seeds containing luminal carbohydrate recognition domains [110]. In humans and other mammals there are four L-type lectins, the first with an intermediate compartment of 53 kDa (ERGIC-53), the second ERGIC-53-like (ERGL), the third being a vesicular integral membrane protein of 36 kDa (VIP36), and the fourth considered VIP36-like (VIPL) [111,112,113,114]. These type I membrane proteins are involved in glycoprotein sorting, trafficking and targeting in luminal compartments of animal cells [115,116] also assisting in the blood coagulation process [117,118,119,120,121]. LTLs therefore have an important role as pattern recognition receptors in the immune system. LTLs can be induced after bacterial infection with lipopolysaccharides (LPS) and peptidoglycans (PGN). These lectins can agglutinate bacteria in a calcium-dependent manner or by opsonization, binding to the surface glycoconjugates of these microorganisms [98,99,122].

The C-type lectin family includes a superfamily of proteins containing the C-type lectin domain (CTLD). Largely described in the Metazoa group, today this group is also associated with sequences from other Eukarya and species of bacteria and viruses. This group encompasses the most diverse category of lectins, with several sub-groups described. The lectin C subgroups include at least 17 classes of proteins [123]. These proteins have functions from the most basal and common role—Ca^2+^-dependent carbohydrate binding—to the most developed roles, such as specifically recognizing protein, lipid and inorganic ligands [123]. C-type lectin receptors (CLRs) are a group of pattern recognition receptors (PRRs) that recognize carbohydrate structures in microbes as pathogen-associated molecular patterns (PAMPs). They are expressed mainly in dendritic cells (DCs) and macrophages [124].

Furthermore, the S-type lectin group was replaced by the galectin group, composed of structural homologues of CRDs, which typically bind glycoconjugates containing beta-galactose. These proteins are mainly expressed in cells from the immune system and in the epithelial cells. Galectins are involved in the recognition of endogenous carbohydrate ligands and also in the binding of glycans on the surface of microorganisms [125]. Galectins present a significant role in the host’s defense against bacteria [90,91].

Finally and no less importantly, the sialic-recognizing Ig-superfamily lectins (Siglecs) constitute the major subfamily of the I-type lectin group (proteins presenting an immunoglobulin (Ig)-like domain that mediates the glycan recognition) [126]. Siglecs have been associated with intracellular signaling and immune response [126,127] and are proposed as a novel pharmacological strategy for immunotherapy and glycotherapy due to their action on cell death, anti-proliferation effects and other cell activities [128]. Siglecs are involved in the host-pathogen interaction, in which the protein was observed as mediating the recognition of sialylated glycans expressed in the lipooligosaccharides [129] and inducing rapid proinflammatory cytokine and type I IFN responses to *Campylobacter jejuni* [130]. Siglecs are positive and negative regulators of the immune system and are reported as being involved in resistance and/or susceptibility to pathogens [131].

## 4. Lectin Structural Analyses

**Figure 1:**
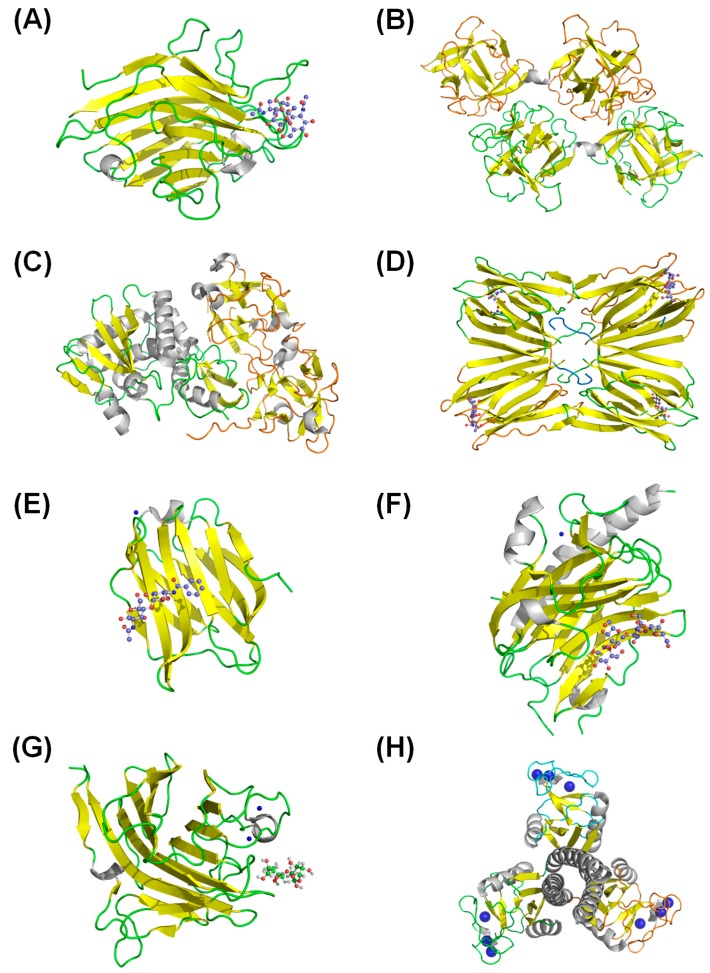
Lectin tridimensional structures in (**A**) arcein from *Phaseolus vulgaris* L. (pdb: 1ioa) [152]; (**B**) amaranthin from *Amaranthus caudatus* (pdb: 1jly) [138]; (**C**) ricin from *Ricinus communis* (pdb: 3rti) [140]; (**D**) jacalin from *Artocarpus integer* (pdb: 1m26) [142]; (**E**) galectin-3 from *Homo sapiens* (pdb: 2xg3) [145]; (**F**) calreticulin from *Mus musculus* (pdb: 3o0w) [147]; (**G**) *Homo sapiens* L-type (pdb: 4gkx) [150] and (**H**) *Homo sapiens* C-type (pdb: 1pwb) [151]. The colors yellow and white represented the secondary structures of β-sheet, α-helices in lectins, respectively. In addition, blue represents the Ca^2+^ ion and carbohydrates are represented in ball and stick.

The plant lectins are constituted by several families which have very conserved recognition sites. Several lectin structures have already been solved by X-ray diffraction, among which ConA from *Canavalia virosa* is a typical prototype. ConA lectin crystallization was previously described in 1936, being the first hemagglutinin isolate with high toxic activity evaluated in rabbits [132]. Furthermore, other lectins have already been elucidated, such as ricin, a lectin from *Ricinus communis*, which exhibited a combination of α-helices and β-sheets [2]. Oliveira and coworkers [133] also demonstrated a similar scaffold for a lectin purified from *Caesalpinia tinctoria* [133]. In most, the tertiary structure conformation of lectins presents structurally dominant β-sheets combined with the presence of α-helices that can also be absent. In spite of lectins’ wide structural diversity, at least one specific site for the carbohydrate link in each polypeptide chain was observed. Lectin-carbohydrate interactions present a carbohydrate recognition domain which might be localized on the surface of the protein molecule, as observed in lectins from Fabaceae [134]. In the active site for carbohydrate ligand the interaction patterns are guided by hydrogen bonds needed for lectin-carbohydrate complex formation. Several studies about the specificity of carbohydrate and plant lectins have revealed that most do not present affinity to monosaccharides. This lesser affinity may also be observed in some red algal lectins which bind to glycoproteins, having no requirement for divalent cations and presenting low molecular masses [135]. Furthermore, plant lectins are commonly encountered in monomeric, dimeric, trimeric and tetrameric forms. For example, monomeric forms of lectins were observed in *Hevea brasiliensis* and in arcein from *Phaseolus vulgaris* L. (Figure 1A) [136,137]. Dimeric lectins are also observed, and these are subdivided into homo- and heterodimeric. Homodimerism is seen in *Erythrina speciosa* Andr. and *Amaranthus caudatus* lectin (amaranthin) (Figure 1B) [60,138]. Conversely, *Ricinus communis* demonstrated two different chains with subunits of 29.5 and 28.5 kDa bonded by one disulfide bond [139]. This heterodimeric lectin, also called ricin, consists of two subunits, namely the A chain showing hydrolitic activity linked to a B chain, involved in carbohydrate binding (Figure 1C) [140]. Trimeric lectins are observed in the red marine alga, *Ptilota filicina*, which presents molecular masses of around 56.9 kDa by gel filtration divided into three subunits of 19.3 kDa, indicating three identical subunits [141]. In turn, jacalin presented a tetramer structure, which also has four lectin domains (Figure 1D) [142]. Another interesting structural feature indicates that carbohydrate binding activity could be improved in the presence of some ions. This common facet observed in the lectin group demonstrated the presence of two important ions, Ca^2+^ and Mn^2+^, which are essential for sugar binding. The amino acids involved in ion binding are highly conserved throughout legume lectins. L-type lectins, for example, present an oligomeric structure in which the monomers consist of antiparallel β-sheets disposed in flat strands. Some of these lectin groups demand Ca^2+^ and transition metal ions (usually Mn^2+^) for their carbohydrate-binding activity [143]. Kabir and coworkers [144], for example, revealed that lectin from *Pisum sativum* L. presents interesting pharmacological activities and is of great interest in biomedical research. Recent studies demonstrated that pea lectin inhibited Ehrlich ascites carcinoma cells. In addition, this lectin also demonstrated activity* in vivo*, causing 63% and 44% growth inhibition of the carcinoma cells in mice when administered at 2.8 and 1.4 mg/kg/day, respectively [144].

In contrast, animal lectins have been elucidated according to their 3D structure. Galectins present a similar CRD, which is folded into two antiparallel β-sheets that are slightly curved, forming a concave ligand-binding site (PFAM: 00337—Galactoside-binding lectin, Figure 1E) with specificity for glycans containing β-galactoside [145]. Calnexin and calreticulin present two distinct structural regions: a globular lectin domain and a proline-rich P-domain inserted between the C- and N-terminal domains [146]. The lectin domain of both these proteins (CNX and CRT) is composed of two curved β-sheets, which form a β-sandwich, similar to the legume lectin domain (Figure 1F) [147]. Moreover, these proteins also present a calcium-binding site, which seems to be mainly involved with structural requirements rather than in the ligand binding [148]. The biological activity in the presence of divalent ions is explained due to structural modification that its ions promoted in the lectins, causing recognition in the carbohydrate, stabilizing the ligand site and directing the amino acid’s spatial position in the carbohydrate ligand. In contrast, the lectins that do not need ions already present the correct structural conformation for carbohydrate recognition [149]. Additionally, L-type animal lectins (Figure 1G) also present a legume-like lectin domain and calcium binding. However, in contrast with the calreticulin, the structural characterization of the *Homo sapiens* LMAN1 protein indicates a correlation between the calcium-binding and the lectin domain activity, in which the ligand could be released with changes in the Ca^2+^ concentration [150]. The calcium requirement is also strong when correlated with the animal C-type lectin family. The human lung surfactant protein D presents three calcium-dependent lectin domains bonded by an α-helical coiled-coil neck (Figure 1H) [151].

## 5. Biotechnological Potential

The use of a myriad of lectins as biotechnological tools has been presented by many researchers in recent years, especially due to their enormous ability to discriminate sugars with high specificity. Plant and animal lectins show rich hydrophobic amino acid regions composed of deep pocket forms. Such hydrophobic pockets allow a number of interactions between lectins and distinct carbohydrates, conferring multiple functions that include the antimicrobials described here [42,153,154,155]. Studies with 42 plant lectins carried out by Barre and coworkers [156] revealed that 32% of the hydrophobic pockets are identical, suggesting a clear structural conserved conformation [156]. Nevertheless, the development of antibiotics from lectins is a real challenge, since their production is expensive and complicated for chemical synthesis due to the complex structural scaffold and the high number of disulphide bonds. One option is the production of heterologous systems, including bacterial vectors such as *E. coli* or *B. subtilis*, or yeast vectors such as different Pichia species. However, since there are lectins that showed bactericidal or fungicidal effects, a selective strategy must be performed in order to prevent the activity of antimicrobial lectins against the host expression vector [157].

Another option consists of producing these lectins by using genetically modified plants [157]. Agricultural losses are a challenging economic and food security problem. Global food security is threatened by population growth and the emergence and spread of crop pests, which is significantly increasing with climate change [158]. Strategies to overcome the damage caused by pathogens include chemical treatment, conventional breeding and transgenic approaches [159]. Beginning in 1980, new strategies for pest control, such as integrated pest management (IPM) and the use of transgenic crops have been proposed and tested, to avoid these crop production losses. Transgenic plants expressing lectin genes have been shown to confer resistance against nematodes and insects. The expression of lectins acts in partial resistance to nematodes in transgenic plants [23,160] and they have the capacity to reduce galling caused by nematodes when these are introduced in the soil [23,161,162]. The exact mechanism of action of lectins against nematodes has not been well elucidated.

The insecticidal activity of different lectins has also been observed [163,164]. The effects of lectins when ingested by insect larvae are in growth inhibition, reduction of size and weight gain, interference in the fecundity of the female, as well as in reducing pupation and the percentage of adult emergence, increasing the total development time, and in some cases resulting in the death of the insect larvae [165,166,167,168,169]. Morover, some transgenic plants expressing lectins also show some resistance against multiple pathogens [49,170]. In this case, a soybean lectin (SBL) was introduced into tobacco plants via Agrobacterium-mediated transformation, improving resistance to infection by *Phytophthora nicotianae* [163]. Another example is the expression of the agglutinin gene from *Pinellia ternata* in tobacco chloroplasts [171]. Such genetic engineering has conferred wide resistance against whitefly, aphids, Lepidopterans and bacterial and viral pathogens. The virus infections have also been focused as a target for transgenic plants synthesizing lectins. Heterologous expression of a jacalin-type lectin (JAX1) in *Nicotiana benthamiana* was capable of interfering with potexvirus infection [39].

Therefore, lectins can be used not only in transgenic plant development. There are some reports describing the use of different lectins for evaluation of cell surfaces [172,173], for blood typing [174,175], as mitogenic agents [176,177,178,179,180], to detect changes in cellular transformation [181,182], in the clearance of sulfated glycoprotein hormones, control of glycoprotein biosynthesis, cell-cell interactions in the immune system [183] and associated with susceptibility and severity in a wide variety of infectious and autoimmune diseases [184,185]. Moreover, lectins can also be applied in laboratory and chemical analyses [173,178,186], since they are easily immobilized on inert supports [173,187].

In the antitumor field *Helix pomatia* agglutinin (HPA), initially isolated from Roman snails, has been used extensively in histopathology, since its binding to tissue sections from breast and colon cancers is correlated with the worst prognosis for patients. HPA recognizes α-D-*N*-acetylgalactosamine (α-Gal-NAc) containing epitopes which are only present in cancer cell lines. The crystal structures of the lectin complexed with two Gal-Nac-containing epitopes associated with cancer, the Tn (α-Gal-NAc-Ser) and Forssman (α-Gal-NAc-1-3-Gal-NAc) antigens, show that lectin’s specificity for GalNAc is due to a particular network of hydrogen bonds. A histidine residue makes hydrophobic contact with the aglycon, rationalizing the preference for GalNAc bearing an additional sugar or amino acid in the alpha position [188]. Furthermore, *Mus musculus* galectin-1 (MMG) isolated from mouse has been shown to attenuate experimental acute and chronic inflammation. MMG recognizes β-galactoside (β-Gal), and experiments have demonstrated that it may be part of a novel anti-inflammatory loop [189].

## 6. Conclusions

In summary, lectins from animal and plant sources have a remarkable repertoire of compounds that not only bind to carbohydrates, but also open novel and unusual possibilities for biotechnology. They are indeed a promising source of natural tools that could bring several benefits to agribusiness and human health. In this context, a combination of bioprospection, structural and molecular biology and genetic engineering will bring real discoveries in the near future.
